# Genome-wide microRNA profiles identify miR-107 as a top miRNA associating with expression of the CYP3As and other drug metabolizing cytochrome P450 enzymes in the liver

**DOI:** 10.3389/fphar.2022.943538

**Published:** 2022-08-17

**Authors:** Marwa Tantawy, Joseph M. Collins, Danxin Wang

**Affiliations:** Center for Pharmacogenomics, Department of Pharmacotherapy and Translational Research, College of Pharmacy, University of Florida, Gainesville, FL, United States

**Keywords:** cytochrome P450, drug metabolism, microRNA, estrogen receptor alpha (ERα), gene expression, CYP3A4, miR-107

## Abstract

Cytochrome P450 (CYP) drug metabolizing enzymes are responsible for the metabolism of over 70% of currently used medications with the CYP3A family being the most important CYP enzymes in the liver. Large inter-person variability in expression/activity of the CYP3As greatly affects drug exposure and treatment outcomes, yet the cause of such variability remains elusive. Micro-RNAs (miRNAs) are small noncoding RNAs that negatively regulate gene expression and are involved in diverse cellular processes including metabolism of xenobiotics and therapeutic outcomes. Target prediction and *in vitro* functional assays have linked several miRNAs to the control of CYP3A4 expression. Yet, their co-expression with CYP3As in the liver remain unclear. In this study, we used genome-wide miRNA profiling in liver samples to identify miRNAs associated with the expression of the CYP3As. We identified and validated both miR-107 and miR-1260 as strongly associated with the expression of CYP3A4, CYP3A5, and CYP3A43. Moreover, we found associations between miR-107 and nine transcription factors (TFs) that regulate CYP3A expression, with estrogen receptor alpha (ESR1) having the largest effect size. Including ESR1 and the other TFs in the regression model either diminished or abolished the associations between miR-107 and the CYP3As, indicating that the role of miR-107 in CYP3A expression may be indirect and occur through these key TFs. Indeed, testing the other nine CYPs previously shown to be regulated by ESR1 identified similar miR-107 associations that were dependent on the exclusion of ESR1 and other key TFs in the regression model. In addition, we found significant differences in miRNA expression profiles in liver samples between race and sex. Together, our results identify miR-107 as a potential epigenetic regulator that is strongly associated with the expression of many CYPs, likely *via* impacting the CYP regulatory network controlled by ESR1 and other key TFs. Therefore, both genetic and epigenetic factors that alter the expression of miR-107 may have a broad influence on drug metabolism.

## Introduction

The cytochrome P450s (CYPs) are comprised of a superfamily of enzymes involved in the biotransformation of xenobiotics and endogenous substances. Members of the CYP1, CYP2, and CYP3 families are the main drug metabolizing enzymes in the liver, responsible for ∼70% of all clinically used medications (Wienkers and Heath, 2005). Among them, the CYP3A subfamily is particularly important in adults because of their broad substrate specificity and prominent expression in liver (Danielson, 2002), where many medications are metabolized. The four *CYP3A* genes (*CYP3A4*, *3A5*, *3A7*, and *3A43*) are encoded in a cluster located on chromosome 7q22.1. CYP3A4 is mainly expressed in the adult liver and is responsible for metabolizing 30%–40% of currently used drugs (Danielson, 2002). CYP3A5 shares many substrates with CYP3A4 and is also expressed outside the liver, but due to the frequent loss of function genetic variant CYP3A5*3, many individuals do not express CYP3A5 (Kuehl et al., 2001). CYP3A7 is primarily expressed in fetal livers with low expression levels observed in adults, while CYP3A43 is expressed in liver and extra-hepatic tissues like testis and prostate, but its substrates remain unclear. Overall, CYP3A-mediated drug metabolism is highly variable between individuals, greatly affecting drug exposure and treatment outcomes.

Many factors are known to influence the expression and function of the CYP3A enzymes. The genetic variant CYP3A4*22 (frequency 4%–8% in Whites and <1% in Blacks) reduces CYP3A4 expression 2–6-fold *via* affecting alternative splicing and transcription (Wang et al., 2011; Wang and Sadee, 2016; Collins and Wang, 2020) and is considered the most clinically relevant genetic variant in CYP3A4, associating with many phenotypes related to CYP3A4 metabolism (Mulder et al., 2021). Besides cis-acting genetic polymorphisms, the expression of the CYP3As is also regulated by trans-acting transcription factors (TFs), epigenetic modifications, and non-genetic factors (Zanger and Schwab, 2013). At the transcriptional level, the expression of many liver enriched TFs is correlated with the expression of CYP3As and other CYPs (Yang et al., 2010). The most significantly correlated TFs include: NR1I3 (constitutive androstane receptor or CAR), RXR (retinoid X receptor), NR1I2 (pregnane X receptor or PXR), HNF4A (hepatocyte nuclear factor 4 alpha), PPARA (peroxisome proliferator activated receptor alpha), FOXA2 (forkhead box A2), AHR (aryl hydrocarbon receptor) and ARNT (aryl hydrocarbon receptor nuclear translocator), as well as our recently identified master regulator ESR1 (estrogen receptor alpha) (Wang et al., 2019; Collins and Wang, 2021a). Moreover, ESR1 appears to be the top regulator for the expression of the CYP3As and other CYP enzymes, explaining up to 63% of the variability in expression of CYP3A4 and the other CYPs (Collins and Wang, 2021a). The activity of these TFs is known to be influenced by endogenous ligands and xenobiotic inducers, and it is likely that underlying genetic and/or epigenetic factors that alter their expression will also contribute to variation in *CYP3A* expression (Zanger and Schwab, 2013).

Micro-RNAs (miRNAs) are small noncoding RNAs of 21–25 nucleotides (nts) that negatively regulate gene expression. This effect is typically mediated by their binding to the 3′-untranslated regions (3′-UTRs) of target mRNAs, thereby interfering with mRNA translation or leading to degradation of the mRNA. There is also evidence for transcriptional control of gene expression *via* miRNA-targeting (Catalanotto et al., 2016). Through these mechanisms, miRNAs play an essential role in gene regulation and have been well-documented for a variety of biological processes, including: differentiation, development, proliferation, apoptosis, and necrosis (Londin et al., 2015; Yang et al., 2017). miRNAs also regulate the expression of genes related to pharmacodynamics and pharmacokinetics (Rieger et al., 2013), thereby altering drug toxicity (Wang et al., 2009; Szkolnicka et al., 2016) and therapeutic outcomes (Mishra et al., 2007; Sharma et al., 2020). Moreover, the expression of miRNAs is subject to change under drug treatment (Gufford et al., 2018) or environmental chemical exposure (Hou et al., 2011), which in turn regulates the expression of the CYP enzymes (Li et al., 2019). The interplay between dysregulation of miRNAs, changes in CYP expression, and CYP-dependent bioactivation is considered to be the underlying cause for environmental toxicology and carcinogenesis (Kalscheuer et al., 2008; Li et al., 2019). However, the regulatory roles of miRNAs in regulating constitutive expression of the CYP enzymes remain unclear.

A few studies have reported miRNA-mediated regulation of the CYP enzymes, including CYP3A4, by either direct 3′UTR binding or through indirect targeting of key TFs controlling CYP expression (Pan et al., 2009; Li et al., 2019). These previous studies focused on several candidate miRNAs that were predicted to bind CYP3A4 or its regulators and used *in vitro* cell models to evaluate the function of the miRNAs on their targets (Li et al., 2019). However, the relationship between the expression levels of these miRNAs and CYP3A4 has not been tested in the liver, with the exception of a single study illustrating the relationship between four miRNAs in a small (27 sample) liver cohort from the Chinese Han population (Wei et al., 2014). Therefore, the role of miRNA-mediated regulation of the *CYP3A* genes in human livers warrants further investigation. In this study, we applied an untargeted genome-wide approach to profile miRNA expression in a large cohort of human liver samples from both European and African American donors to search for miRNAs that may regulate the expression of the CYP3As.

## Materials and methods

### Human liver samples

Human liver samples from African American (AA) (*n* = 104) and European American (EA) (*n* = 127) donors were obtained from The Cooperative Human Tissue Network (CHTN). Demographics of liver donors are in Supplementary Table S1. The median ages are AA—56 years (range 0–97) and EA—61 years (range 14–83) and the percentage of males for each are AA—44% and EA—46%. The University of Florida Institutional Review Board approved the human tissue study.

### RNA preparation and quantitative gene expression analysis

Total RNA was extracted from the liver tissue samples using the Direct-zol RNA miniprep plus kit (ZYMO Research, United States) followed by cDNA synthesis as described previously (Collins and Wang, 2021a). mRNA levels of CYP3As, CYP2Cs, CYP1As, ESR1, NR1I3, NR1I2, RXR, HNF4A, PPARA, FOXA2, AHR, and ARNT, and the internal control β-actin were measured with real-time PCR using TaqMan assays (Thermo Fisher Scientific) or SYBR Green with gene-specific primers as described (Collins and Wang, 2021a). The relative expression of each gene was calculated using the following formula: expression level of tested gene = antilog2 (mean Ct value of internal control—Ct value of tested gene) *10^6^. After Log_10_ transformation, the expression data of all genes tested followed normal distribution as described previously (Collins and Wang, 2021a). The expression profiles of 12 CYPs and 9 TFs, the relationship between each CYP, the association between CYP and TFs, and the racial differences in expression between AA and EA have been described previously (Collins and Wang, 2021a).

### miRNA profiling in the discovery phase

We used the TaqMan OpenArray Human MicroRNA panels and QuantStudio 12K Flex (Applied Biosystem, Thermo Fisher Scientific, CA, United States) for initial genome wide miRNA profiling in 96 liver samples (48 AA and 48 EA). The TaqMan OpenArray Human MicroRNA Panel contains 754 miRNAs with validated TaqMan miRNA assays derived from Sanger miRbase release v.14. Total RNA (10 ng) was reverse-transcribed using MegaplexTM pool RT-primers. Pre-amplification of cDNA with MegaplexTM PreAmp primers was performed according to the manufacturer’s protocol and recommendations (Thermo Fisher Scientific, United States). The pre-amplified products were diluted and mixed with TaqMan OpenArray Real-time PCR master mix and added to 384-well OpenArray Sample Loading plates. TaqMan OpenArray Human MicroRNA panels were automatically loaded by the AccuFill System and then placed in QuantStudio TM 12K Flex Real-Time PCR system for cycling.

### Open array data quality control and statistical analysis

Cycle threshold (Ct) values with amplification scores (Amp Scores) below 1.24 and Cq confidence (Cq Conf) < 0.8 were filtered out. miRNAs with missing Ct values or Ct > 35 in >50% samples and samples with low miRNA detection were excluded. Data was normalized using global normalization by subtracting the individual Ct from the mean Ct of all miRNAs (Luo, 2012). Differential expression of miRNAs between race and sex was detected using the 2^–ΔΔCT^ method (Livak and Schmittgen, 2001) and differences that exceeded 2-fold (FC > 2 or FC < 0.5) and *p* value ≤ 0.05 were considered statistically significant. Linear regression was used to test the association between the miRNAs and CYP3A expression, adjusting for race, sex, and age. All statistical analyses were carried out in the statistical programming environment R, using SAS v9.4 (Cary, NC) and Statistical Package for the Social Sciences SPSS v.26 (IBM Corp., United States).

### miRNA targeted TaqMan assays for validation and data analysis

The TaqMan Advanced miRNA assays (Thermo Fisher Scientific, United States) were used to determine the expression of the two most significant miRNAs (miR-107 and miR-1260) in 231 liver samples for validation. Total RNA (10 ng) was reverse transcribed and quantitative real-time PCR was performed according to the manufacturer’s protocol and recommendations (Thermo Fisher Scientific, United States). From the discovery data set, we found that the combination of miR-132 and miR-484 was the most appropriate internal control for data normalization, as they had the most stable value of 0.003 according to Normfinder software (Andersen et al., 2004). Thus, the expression levels of miR-132 and mir-484 were also measured for normalization purposes. A multiple linear regression model was used to test the association between miRNAs and the expression of the CYP3As. We used forward and backward stepwise regression to select the best set of predictors in the multiple linear regression models with a cut-off *p*-value ≤ 0.05. Covariates considered for inclusion were: sex, race, age, and genotypes (CYP3A4*22 for CYP3A4 and CYP3A5*3 for CYP3A5, respectively), and nine TFs known to regulate CYP3A gene expression (Collins and Wang, 2021a). Modulated modularity clustering (MMC) (Stone and Ayroles, 2009) was used to explore the inter-miRNA relationships. MMC was designed to detect latent structure of the variance-covariance matrix using weighted graphs. The method searches for optimal community structure and detects the magnitude of pairwise relationships. The optimal number of clusters and the optimal cluster size were selected by using Spearman correlation.

### Genotyping assays (rs62471956 and rs776746)

The single nucleotide polymorphism rs776746 (CYP3A5*3) and rs62471956, which is in complete linkage disequilibrium (LD) with rs35599367 (CYP3A4*22) (Collins and Wang, 2020), were genotyped using the OpenArray genotyping platform according to the manufacturer’s protocol (Life Technology, CA, United States).

### Quantification of CYP3A4 protein in liver samples

CYP3A4 protein levels in 154 liver samples were measured using the capillary western blotting Jess system as described (Collins and Wang, 2021b). Briefly, total tissue lysates were prepared (Collins and Wang, 2021b) and a mouse anti-CYP3A4 antibody (R&D MAB 9079, 1:20 dilution) and NIR-conjugated anti-mouse secondary antibody (Biotechne, San Jose, CA, United States, 1:20 dilution) were used for detection. The total protein loaded in each lane was measured using the total protein channel in the Jess system. CYP3A4 protein (pmol) per mg total protein was calculated from a standard curve generated from a purified GST-fusion CYP3A4 protein (FisherScientific) measured on the Jess system. After log10 transformation, CYP3A4 protein levels followed a normal distribution as previously described (Collins and Wang, 2021b).

## Results

### Genome-wide expression profile of miRNAs in human liver samples

A total of 252 miRNAs in 91 samples (AA = 46, EA = 45) passed quality control. Three internal controls (U6rRNA, RNU44, and RNU48) and two miRNAs predicted to be tRNA fragments (HSA-MIR-1274B and HSA-MIR-720) were excluded, resulting in 247 miRNAs being included for further analysis. The expression levels between the 247 miRNAs varied drastically, with median Ct values ranging from 14.3 to 30.1 (>32,000-fold difference) (Supplementary Figure S1). Supplementary Table S2 lists the top 20 highest and lowest expressed miRNAs. Of these, miR-122, miR-24, and miR-19 showed the highest expression levels in liver, consistent with previous results (Rieger et al., 2013).

There was also significant variation in the expression of the miRNAs between the 91 samples. The inter-quantile range (IQR) for the Ct values ranged from 0.28 to 14.24 (Supplementary Figure S2). Nearly 60% (*n* = 149) of the miRNAs showed an IQR < 2 (<4-fold difference), while 10% (*n* = 26) of the miRNAs showed an IQR of 5–14 (32–16,384-fold). The miRNAs miR-1260, miR-29c, and miR-215 had the largest IQR of 11–14 (2048–16,384-fold).

To explore the relationship between the expression of the 247 miRNAs we used MMC (Stone and Ayroles, 2009). MMC identified seven clusters (Supplementary Table S3) with the number of miRNA members in each cluster ranging from 11 to 146. Cluster 1 had the strongest correlation between miRNAs (*n* = 15, average correlation = 0.72), while clusters 2–6 showed moderate correlation (*n* = 86, average correlation = 0.49–0.58). The majority of the miRNAs (*n* = 146) were clustered in Cluster 7 and were only weakly correlated (average correlation = 0.17).

**FIGURE 1:**
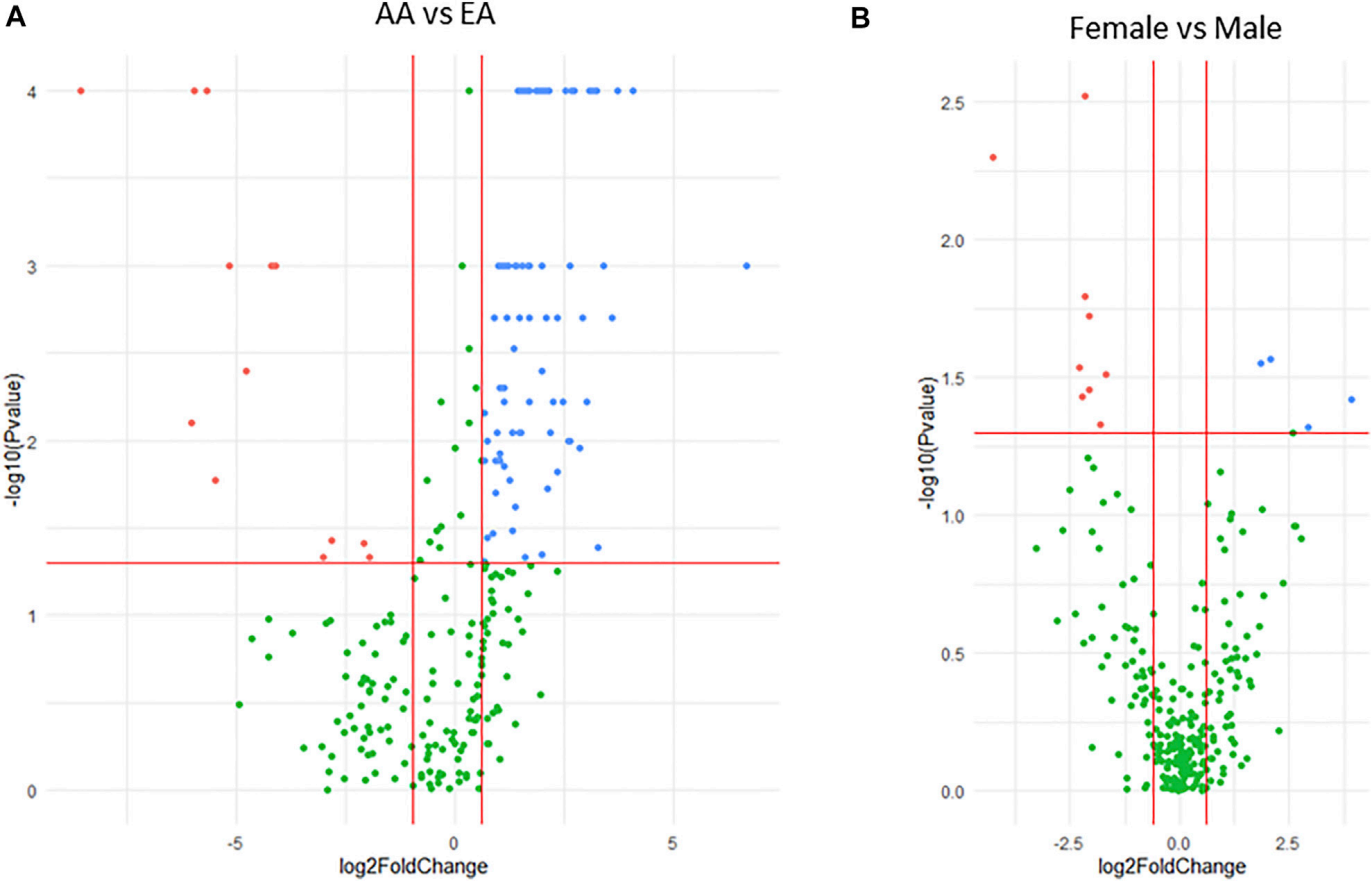
Volcano plots showing differential expression of miRNAs between race **(A)** and sex **(B)** in liver samples. The plots indicate expression of significantly higher (blue dots) and lower (red dots) miRNAs in **(A)** AA vs. EA or **(B)** female vs. male. Negative log10 *p*-values are plotted on the y-axis, and log2 normalized fold change in expression is on the x-axis. For a miRNA to be considered as both significantly and differentially expressed a 2-fold difference and *p* ≤ 0.05 was required.

Expression of the miRNAs differed significantly between samples originating from either AA or EA donors. Thirteen miRNAs were lower in AA compared to EA (fold-change < 0.5, *p* < 0.05) and 79 were higher (fold change > 2, *p* < 0.05) (Figure 1A). Similarly, the expression of the miRNAs differed between males and females, with nine miRNAs being lower (fold-change < 0.5, *p* < 0.05) and five being higher (fold-change > 2, *p* < 0.05) in females (Figure 1B). In addition, two miRNAs were positively correlated with age, while eight were negatively correlated (Supplementary Table S4). Thus, age, sex, and race were included as covariates for further association analyses.

### The association between the expression of miRNAs and the CYP3As

**FIGURE 2:**
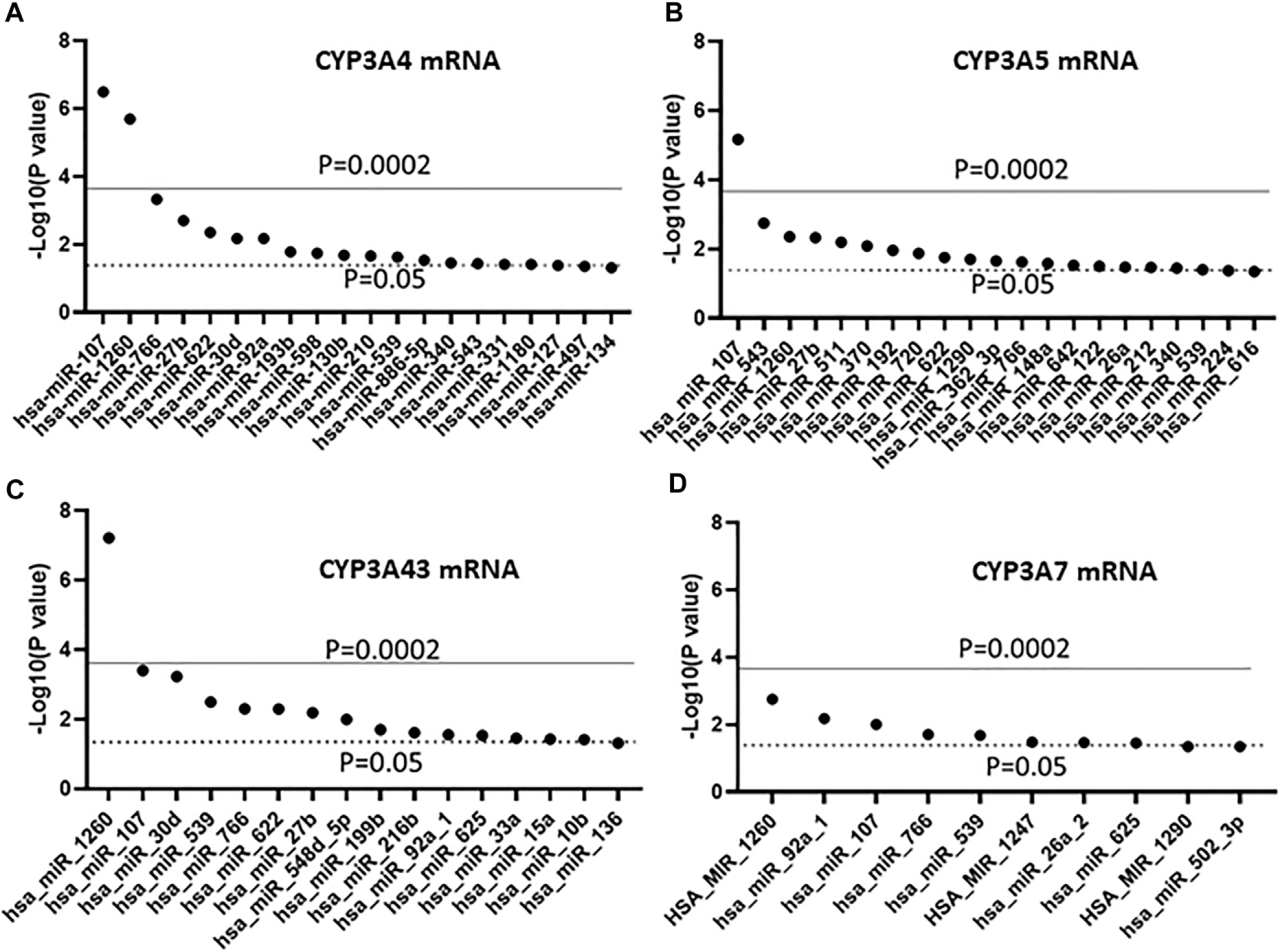
Association between miRNAs and mRNA expression of four *CYP3A* genes in human liver samples. **(A)** CYP3A4; **(B)** CYP3A5; **(C)** CYP3A43 and **(D)** CYP3A7. miRNAs were measured using The TaqMan OpenArray Human MicroRNA panels. Linear regression was used to test the association between the 247 measured miRNAs and mRNA expression of the four CYP3As. Only miRNAs with an association *p* ≤ 0.05 are shown.

In a previous study, we showed mRNA expression for 12 CYP genes in the same liver cohort (Collins and Wang, 2021a). Using that dataset, we tested for association between the expression of the 247 miRNAs and four *CYP3A* genes using linear regression. As shown in Figure 2A, 21 miRNAs were associated with the expression of CYP3A4 (*p* < 0.05), however, after Bonferroni correction, only miR-107 and miR-1260 remained significant (*p* < 0.0002). Similar results were found with CYP3A5 and CYP3A43 (Figures 2B,C), but the associations between CYP3A5/miR-1260 and CYP3A43/miR-107 became insignificant after Bonferroni correction. In contrast, none of the miRNAs showed significant association with CYP3A7 after correction (Figure 2D).

**FIGURE 3:**
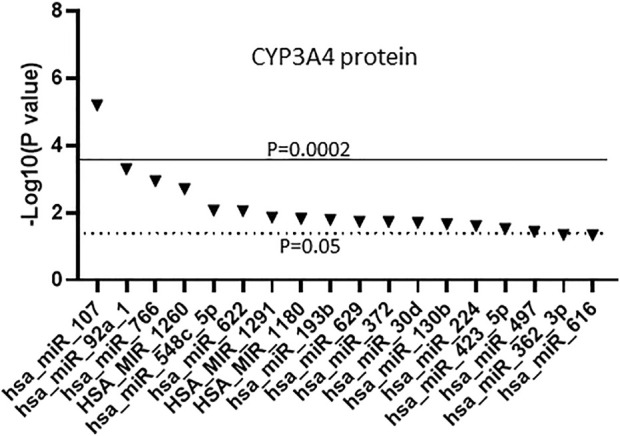
Association between miRNAs and CYP3A4 protein expression in human liver samples. miRNAs were measured using The TaqMan OpenArray Human MicroRNA panels. Linear regression was used to test the association between the 247 measured miRNAs and CYP3A4 protein expression. Only miRNAs with an association *p* ≤ 0.05 are shown.

Since miRNAs can regulate gene expression *via* transcriptional or post-transcriptional pathways we also tested for association between the miRNAs and CYP3A4 protein. Previously, we reported that in the 179 human liver samples the average protein concentration is 101 pmol/mg, with a range of 6.9–257 pmol/mg, and that CYP3A4 mRNA and protein levels are moderately correlated (correlation coefficient = 0.778) (Collins and Wang, 2021b). We associated the expression of the miRNAs to the previous CYP3A4 protein dataset and found 18 miRNAs that were significantly associated (*p* < 0.05) with CYP3A4 protein levels (Figure 3), nine of which (miR-107, miR-1260, miR-766, miR-622, miR-30d, miR-92a, miR-193b, miR-1180 and miR-497) were also associated with CYP3A4 mRNA as shown in Figure 2. Notably, miR-107 was also found to be the most significant miRNA associated with CYP3A4 protein levels. Conversely, miR-1260 was not found to be significantly associated with CYP3A4 protein expression after Bonferroni correction.

### Validation of the association between miR-107/miR-1260 and the CYP3As

The genome-wide approach identified miR-107 and miR-1260 as the top two miRNAs associated with the expression of CYP3A4, CYP3A5, and CYP3A43 in human liver samples. To validate these results, we measured the expression of both miR-107 and miR-1260 using targeted TaqMan assays in our entire liver sample cohort (*n* = 231). After normalization by an internal control (combination of miR-132 and miR-484, see Methods), the expression levels of miR-107 and miR-1260 followed a normal distribution (Supplementary Figure S3). We then tested the association between these miRNAs and CYP3A expression. As expression of several miRNAs was affected by race, sex, and age, we included these covariates in our association models. Also, two known variants that reduce expression of the CYP3As, CYP3A4*22 (Wang et al., 2011) and CYP3A5*3 (Kuehl et al., 2001) were included to account for their impact on CYP3A expression. In agreement with our results from the discovery phase, miR-107 and miR-1260 were negatively associated with the expression levels of CYP3A4, CYP3A5, and CYP3A43, but not CYP3A7 (Table 1).

**TABLE 1:** Association between the expression levels of miR-107/miR-1260 and the *CYP3A* genes in human liver samples (*n* = 231) using linear regression analysis.

	miR-107		miR-1260	
Gene	Estimate ± SE	*p*-value	Estimate ± SE	*p*-value
CYP3A4 un-Adjusted	−0.270 ± 0.047	3.54E-08***	−0.073 ± 0.046	0.115
CYP3A4^a^	−0.266 ± 0.048	1.08E-07***	−0.106 ± 0.051	0.038*
CYP3A4^b^	−0.064 ± 0.034	0.05863	−0.029 ± 0.032	0.353
CYP3A5 un-Adjusted	−0.174 ± 0.043	8.53E-05***	−0.037 ± 0.041	0.368
CYP3A5^a^	−0.173 ± 0.035	1.92E-06***	−0.081 ± 0.036	0.028*
CYP3A5^b^	−0.029 ± 0.022	0.19361	−0.024 ± 0.021	0.261
CYP3A7 un-Adjusted	−0.104 ± 0.063	0.102	0.102 ± 0.058	0.082
CYP3A7^a^	−0.107 ± 0.064	0.0939	0.102 ± 0.059	0.083
CYP3A7^b^	−0.023 ± 0.064	0.725	0.075 ± 0.058	0.198
CYP3A43 un-Adjusted	−0.352 ± 0.056	2.23E-09***	−0.150 ± 0.055	0.007*
CYP3A43^a^	−0.345 ± 0.054	9.51E-10***	−0.127 ± 0.053	0.018*
CYP3A43^b^	−0.077 ± 0.034	0.023*	−0.060 ± 0.029	0.043*

a Adjusting for covariates age, gender, race, and genotype, rs62471956 (for CYP3A4) or rs776746 (for CYP3A5).

b Adjusting for covariates age, gender, race, rs62471956 or rs776746 genotype, and transcription factors selected using forward and backward stepwise regression. The transcription factors included in the final model—CYP3A4: ESR1, ARNT, NR1I3, PPARA, AHR; CYP3A5: RXRA, ESR1, R1I3, AHR, FOXA2; CYP3A7: RXRA, ESR1, NR1I2; CYP3A43: ESR1, NR1I3.

*p value <0.05; **p value < 0.001; ***p value < 0.0001.

As expression of the CYP3As is known to be controlled by multiple TFs, we also tested for association between miR-107 and miR-1260 and the expression of nine key CYP3A TFs (ESR1, NR1I3, NR1I2, RXR, HNF4A, PPARA, FOXA2, AHR, and ARNT) that correlate with expression of the CYP3As (Collins and Wang, 2021a). Forward and backward stepwise regression (cut-off *p* ≤ 0.05) was used to select which TFs should be included in the multiple linear regression models, which varied for the different CYP3A members (Table 1). Interestingly, when the selected TFs were included in the models, the association between either miR-107 or miR-1260 with the CYP3As either became insignificant (CYP3A4 and CYP3A5) or much less significant (CYP3A43) (Table 1).

Consistent with our results showing miR-107 association with CYP3A4 mRNA, miR-107 is associated with CYP3A4 protein levels after adjusting for age, sex, race, and CYP3A4*22 genotype, and the strength of association is reduced after further adjusting for TFs (Table 2). Also, there was no association between expression levels of miR-1260 and CYP3A4 protein, in agreement with our genome-wide association results.

**TABLE 2:** Association between the expression levels of miR-107/miR-1260 and CYP3A4 protein levels in human liver samples (*n* = 154) using linear regression analysis.

	miR-107		miR-1260	
Gene	Estimate ±SE	*p*-value	Estimate ±SE	*p*-value
CYP3A4 un-Adjusted	−0.1113 ± 0.026	4.35E-05***	−0.0099 ± 0.027	0.713
CYP3A4^a^	−0.1144 ± 0.027	3.77E-05***	−0.0181 ± 0.029	0.538
CYP3A4^b^	−0.067 ± 0.023	0.0049*	0.0001 ± 0.023	0.996

a Adjusting for covariates age, gender, race, and rs62471956 genotype.

b Adjusting for covariates age, gender, race, rs62471956 genotype, and transcription factors (ESR1, NR1I3, and FOXA2) selected using forward and backward stepwise regression.

*p value <0.05; **p value < 0.001; ***p value < 0.0001.

These results indicate that the association between miR-107/miR-1260 and the *CYP3A* genes may be indirectly mediated through their action on TFs. Indeed, miR-107 is negatively associated with all nine TFs tested in this study, and miR-1260 is also negatively associated with the expression of NR1I3, NR1I2, FOXA2, and HNF4A (Table 3). As many of these TFs also regulate the other CYP enzymes (Wang et al., 2019; Collins and Wang, 2021a), we also tested for association between miR-107/miR-1260 and the other nine drug-metabolizing CYPs. As expected, miR-107 is negatively associated with all nine CYP enzymes in the CYP1 and CYP2 families (Table 4) after adjusting by age, sex, and race. Similar to what we observed for the CYP3As, when TFs were included in the regression model, the association between miR-107 and the CYPs became either less significant or insignificant. miR-1260 was only associated with the expression of the CYP3As, CYP1A1, and CYP1A2, but not the other seven CYP enzymes tested (Supplementary Table S5). After including TFs in the model, the associations between miR-1260 and two CYP1A enzymes also became less significant.

**TABLE 3:** Association between the expression levels of miR-107/miR-1260 and nine transcription factors tested in this study in human liver samples (*n* = 231) using linear regression analysis adjusting for covariates age, race, and sex.

	miR-107		miR-1260	
Gene	Estimate ±SE	*p*-value	Estimate ±SE	*p*-value
ESR1	−0.301 ± 0.053	3.29E-08***	−0.058 ± 0.052	0.265
NR1I3	−0.252 ± 0.042	1.12E-08***	−0.114 ± 0.041	0.00656*
NR1I2	−0.160 ± 0.031	5.29E-07***	−0.114 ± 0.029	1.14E-03**
ARNT	−0.055 ± 0.027	0.0384*	−0.030 ± 0.026	0.254
AHR	−0.087 ± 0.025	4.98E-03**	−0.039 ± 0.025	0.1178
FOXA2	−0.147 ± 0.030	1.63E-06***	−0.067 ± 0.029	0.0204*
RXRA	−0.103 ± 0.031	1.01E-03***	0.009 ± 0.029	0.7554
PPARA	−0.152 ± 0.032	4.07E-06***	−0.042 ± 0.031	0.1773
HNF4A	−0.104 ± 0.031	0.000962**	−0.070 ± 0.029	0.0174*

*p value <0.05; **p value < 0.001; ***p value < 0.0001.

**TABLE 4:** Association between expression levels of miR-107 and nine CYP genes in human liver samples (*n* = 231) using linear regression analysis with or without adjusting for transcription factors as indicated. Age, sex, and race were included as covariates in all analysis.

	Not including TFs			Including TFs			
Gene	Coefficient	SE	*p* value	Coefficient	SE	*p* value	TFs included
CYP1A1	−0.2387	0.0568	3.76E-05***	−0.0999	0.0561	0.076	AHR, NR1I3, ESR1, NR1I2
CYP1A2	−0.2317	0.0449	5.35E-07***	−0.0728	0.0346	0.036*	AHR, ESR1, NR1I3, PPARA, FOXA2
CYP2A6	−0.2357	0.0586	7.78E-05***	−0.0198	0.0454	0.662	ESR1, AHR, NR1I3, PPARA, RXRA
CYP2B6	−0.2383	0.0559	3.01E-05***	−0.0212	0.0426	0.618	ESR1, PPARA, and AHR
CYP2C8	−0.2545	0.0424	7.93E-09***	−0.0357	0.0237	0.137	ESR1, AHR, NR1I3, PPARA, ARNT
CYP2C9	−0.2045	0.0378	1.62E-07***	−0.0253	0.0256	0.324	ESR1, AHR, NR1I3, and PPARA
CYP2C19	−0.1917	0.0425	1.02E-05***	−0.0124	0.0291	0.671	ESR1, AHR, NR1I3, RXRA, PPARA
CYP2D6	−0.2383	0.0559	3.01E-05***	−0.0003	0.029	0.991	NR1I3, ESR1, AHR, and ARNT
CYP2E1	−0.2582	0.0407	1.21E-09***	−0.082	0.0299	0.006*	AHR, ESR1, NR1I3, HNF4A

*p value < 0.05; ***p value < 0.0001.

## Discussion

Using an untargeted genome-wide miRNA expression profiling approach we identified miR-107 and miR-1260 as two miRNAs associating with the expression of CYP3A4, CYP3A5, and CYP3A43 in 91 liver samples (Figure 2). The results were then validated in a larger liver cohort of 231 samples (Tables 1, 2). Furthermore, we found an association between miR-107 and nine TFs known to regulate the expression of the CYP genes (Tables 3, 4). These results indicate a potential broad regulatory role of miR-107 on the expression of CYP enzymes *via* regulating the expression of key CYP controlling TFs. Moreover, we found profound differences in liver miRNA expression between race and sex. To our knowledge, this is the first study to explore genome-wide liver miRNA expression in a large number of samples originating from both European and African Americans and to also compare differences between the two racial backgrounds, gender, and to analyze associations between miRNAs and the *CYP3A* genes.

### Potential regulatory roles of miR-107 and miR-1260 on the expression of CYP3A4 and the other drug metabolizing cytochrome P450 enzymes

Previous miRNA binding site prediction analysis followed by functional studies in cell models has linked 21 miRNAs to the expression of CYP3A4 *via* direct binding to its 3′UTR (Pan et al., 2009; Wei et al., 2014; Shi et al., 2015; Liu et al., 2016; Gill et al., 2017; Yan et al., 2017; Yu D et al, 2018) (Supplementary Table S6). However, it is unknown whether these miRNAs are expressed in the liver and whether their expression levels are correlated with CYP3A4. Probes to detect all 21 previously identified miRNAs were present in the TaqMan OpenArray Human MicroRNA panels used here. However, our study found that 13/21 (62%) were not readily detectable in liver samples and were therefore excluded from our further analyses. Among the eight miRNAs also measured in our samples, only one (miR-27b) was significantly associated with the expression of CYP3A4 mRNA (*p* = 0.035), while two others were significantly associated with CYP3A4 protein levels (miR-548-3p, *p* = 0.0086 and miR-629, *p* = 0.018) (Supplementary Table S6). These results indicate that most of the previously identified CYP3A4-relevant miRNAs play an uncertain role in the liver or that our approach did not readily detect their effects. Based on our results, miR-107 is the most significant miRNA associated with expression of CYP3A4, as well as CYP3A5, CYP3A43, and all nine CYP genes tested. These findings suggest a potential broad regulatory role of miR-107 on the expression of the CYP enzymes, and therefore, drug metabolism.

We also identified and validated miR-1260 as associated with the expression of CYP3A4, CYP3A5, CYP3A43, CYP1A1, and CYP1A2, but not the other seven CYP genes tested. Therefore, compared to miR-107, which appears to play a broad role in CYP regulation, the role of miR-1260 appears to be limited to the CYP3A and CYP1A families. Also, compared to miR-107, the expression level of miR-1260 in the liver is 97-fold lower, the strength of its association with the CYPs is weaker, and there is no association between miR-1260 and CYP3A4 protein levels. Thus, the *in vivo* impact of miR-1260 on CYP expression and drug metabolism may be less significant than miR-107.

### The potential role of transcription factors and estrogen receptor alpha in mediating the association between miR-107 and the cytochrome P450s

Previously published computational analyses using multiple miRNA target prediction algorithms did not identify miR-107 binding sites in the 3′UTR of CYP3A4 (Ramamoorthy and Skaar, 2011; Swart and Dandara, 2014). Therefore, miR-107 regulation of CYP3A4 is unlikely to be mediated by direct binding to its 3′UTR. Instead, based on our results, it appears that miR-107 may regulate CYP3A4 expression indirectly through modulating the expression of key CYP-controlling TFs, as it is negatively associated with all nine TFs tested. Furthermore, inclusion of these TFs in our regression models caused the association between miR-107 and both CYP3A4 mRNA and protein to become insignificant (Tables 1, 2).

Of the key CYP-controlling TFs, the negative effect of miR-107 appears to be strongest for ESR1 (Table 3). Previously, we identified ligand-free ESR1 (i.e., ESR1 function in the absence of estrogen) as a master regulator mediating the expression of CYP3A4 and many other CYPs in hepatocytes and liver tissue (Wang et al., 2019; Collins and Wang, 2021a; Collins et al., 2021). Interestingly, miR-107 is predicted to directly bind to the 3′UTR of ESR1 and caused reduced ESR1 mRNA and protein expression in a cell model (Bao et al., 2019). It seems possible then, that the widespread negative association of miR-107 with the CYPs may be mediated by its direct, negative effect on ESR1. In support of this model, miR-107 is not associated with CYP3A7, which is a fetal enzyme that is not well associated with ESR1 or the other TFs (Collins and Wang, 2021a). Therefore, the effects of miR-107 appear to be limited to CYP enzymes regulated by ESR1 and the other TFs tested here. It is worth mentioning that ESR1 also regulates several of the other CYP-controlling TFs (e.g., NR1I2, FOXA2, HNF4A, and PPARA) (Wang et al., 2019) and may be the underlying reason why these TFs are also associated with miR-107 levels. However, there are likely non-ESR1 mediated miR-107 effects as well. For instance, NR1I3 was strongly associated with miR-107 expression, while ESR1 does not appear to regulate its expression (Wang et al., 2019).

In contrast to its apparent indirect effect on CYP3A4, miR-107 is reported to directly regulate CYP2C8, and potentially all three CYP2Cs, as miR-107 binding motifs are also in CYP2C9 and CYP2C19 (Zhang et al., 2012). Our results showed that all three CYP2C transcripts are strongly and negatively associated with miR-107 and that these associations disappear when including the TFs in the regression models (Table 4). These results indicate that miR-107 may regulate the expression of the CYP2C enzymes through both direct and indirect mechanisms and at both the transcriptional and post-transcriptional levels. As miRNAs have many potential targets, and regulation of the CYPs involves numerous regulatory proteins, the role of miR-107 in CYP regulation warrants further investigation.

Like miR-107, the regulatory role of miR-1260 also appears to be indirectly mediated by TFs, since including TFs in the regression models also abolished or reduced the association between miR-1260 and the CYPs. miR-1260 is associated with the expression of NR1I2, NR1I3, FOXA2, and HNF4A, but not ESR1. Consistently, miR-1260 is most strongly associated with the expression of CYP1A1 and CYP1A2, two CYPs that are only minimally associated with expression of ESR1 (Collins and Wang, 2021a). Therefore, it appears that miR-1260 may have a role in a different CYP regulatory network than miR-107.

### Clinical implications and differences in miRNA profiles between race, sex, and age

miRNA expression is subject to change under many physiological conditions, disease states, and environmental effects (Gulyaeva and Kushlinskiy, 2016), potentially altering downstream pathways in response. miR-107 belongs to a highly conserved miR-15/miR-107 gene family that shares a common “AGCAGC” sequence near the end of the mature miRNAs’ 5′UTR (Finnerty et al., 2010). miR-107 is transcribed from the host gene *PANK1*, which is broadly expressed in many tissues and has been implicated in cancers (Turco et al., 2020), chemosensitivity (Chen et al., 2021), ischemic stroke (Yang et al., 2014), insulin resistance (Yang et al., 2020), and neuronal differentiation (Croizier et al., 2018). Dysregulation of miR-107 is associated with diverse pathways, including: obesity and diabetes (Foley and O'Neill, 2012), Alzheimer’s disease (Chang et al., 2017), cancers (Turco et al., 2020; Chen et al., 2021), and drug treatment and environmental exposure (Chou et al., 2017; Chen et al., 2020). In parallel, CYP substrate drug clearance and expression of the CYP enzymes also change in many of these events, such as obesity (Brill et al., 2012), diabetes (Darakjian et al., 2021), and cancers (Fahy et al., 2012). Whether miR-107 is contributing to altered expression of the CYPs under these adverse conditions is an interesting future direction.

In comparison, relatively little has been reported concerning miR-1260. Recent studies have shown that miR-1260 is upregulated in numerous cancers, including hepatic carcinoma (Yan et al., 2013) and implicated in chemosensitivity (Zhao et al., 2018), and hypoxia-induced vascular smooth muscle cell proliferation (Seong and Kang, 2020). Interestingly, both the CYP3As (Yu et al, 2018b) and CYP1As (McKinnon et al., 1991) are down-regulated in hepatic cancer cells, possibly mediated by increased miR-1260 expression in these cells. Also, in HepaRG cells (hepatic cell line) treatment with the well-characterized CYP3A inducer rifampicin also caused significant reduction in miR-1260 expression (Swart and Dandara, 2019). Rifampicin-induced CYP3A expression is mediated by NR1I2 (PXR) (Chai et al., 2013), which we found to be significantly and negatively associated with miR-1260 expression (Table 4). Therefore, while miR-1260 appears to function in a non-ESR1 regulatory network, its perturbation in response to cancer progression or drug treatment may play a significant role in regulation of the CYPs.

Our results showed large differences in the expression levels of miRNAs between liver samples from AA and EA donors (Figure 1). A previous study also identified 33 miRNAs that are differentially expressed in lymphoblastoid cell lines (LCLs) between residents of Utah (CEU) and Nigerians (YRI), and these differentially expressed miRNAs were significantly and inversely associated with the expression of at least one mRNA, indicating racial differences in miRNA expression may link to phenotypic differences between populations (Huang et al., 2011). Interestingly, of the 33 miRNAs showing racial expression differences in LCLs, 17 are expressed in the liver, 13 of which (76%) also showed racial expression differences in our data, indicating there may be common mechanisms underlying racial differences in miRNA expression across different tissues. Our previous results show that many CYPs and CYP-regulating TFs are differentially expressed between AA and EA (Collins and Wang, 2021a). However, there was no differential expression of miR-107 (*p* = 0.652) or miR-1260 (*p* = 0.192) between AA and EA in this study, indicating that miR-107/miR-1260 are unlikely to contribute to differential expression of the CYPs and TFs between AA and EA.

Sex-biased gene expression is pronounced in the liver (Zhang et al., 2011) and miRNAs may be contributing to these biases. We also found several miRNAs that differ in expression between the sexes (*n* = 13), but miR-107 (*p* = 0.644) and miR-1260 (*p* = 0.323) were not among these. Therefore, it appears that these two miRNAs do not contribute to previously reported sex-biased expression of the CYPs (Wolbold et al., 2003; Lamba et al., 2010).

Differential expression of several miRNAs in the liver has been associated with age (Rieger et al., 2013), and our results identified eight miRNAs to be weakly associated with age (Supplementary Table S4). Compared with the previous study (Rieger et al., 2013), only one miRNA (miR-31) was also identified. The reason for this discrepancy is unknown and requires further investigation. However, some of liver samples in previous study were derived from donors with liver diseases (Rieger et al., 2013) and it is unclear whether disease state may confound miRNA-age correlation.

In conclusion, we have identified both miR-107 and miR-1260 as two top miRNAs associated with the expression of the CYP enzymes. The effect of miR-107 appears to be through alteration of key CYP-regulating TFs, possibly through an ESR1-directed regulatory network. Thus, if validated, expression of miR-107 may serve as an indicator for the expression and activity of many relevant drug-metabolizing enzymes in the liver. Therefore, any physiological, pathophysiological, or environmental conditions that alter miR-107 have the potential to broadly influence drug metabolism and treatment outcomes.

## Data Availability

The miRNA expression datasets presented in this study can be found in Supplementary Table S7.
