# Supplementary material for: Genome-wide microRNA profiles identify miR-107 as a top miRNA associating with expression of the CYP3As and other drug metabolizing cytochrome P450 enzymes in the liver
Source: Front Pharmacol. 2022 Aug 17;13:943538. doi: 10.3389/fphar.2022.943538 (PMC9428441; doi:10.3389/fphar.2022.943538)
Supplement: Supplementary file 1 [file DataSheet1.PDF]

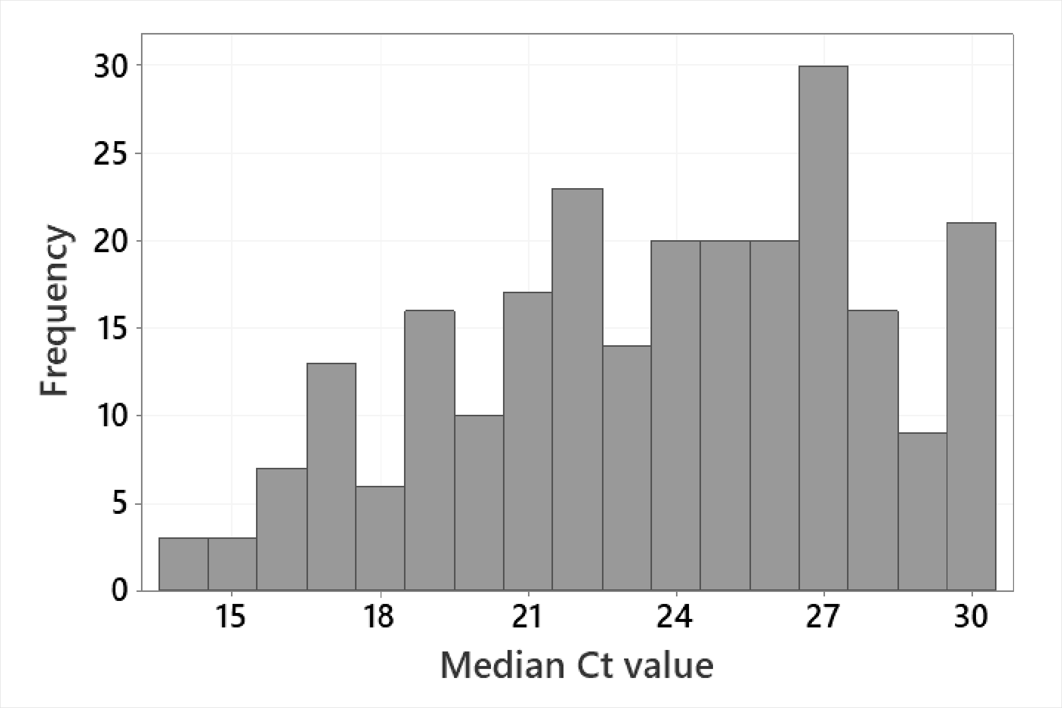

Supplemental Figure 1. Distribution of median Ct values for 247 miRNAs, after excluding three internal controls (U6rRNA, RNU44 and RNU48) and two miRNAs (HSA-miR-1274B and HSA-miR-720) predicted to be tRNA fragments.

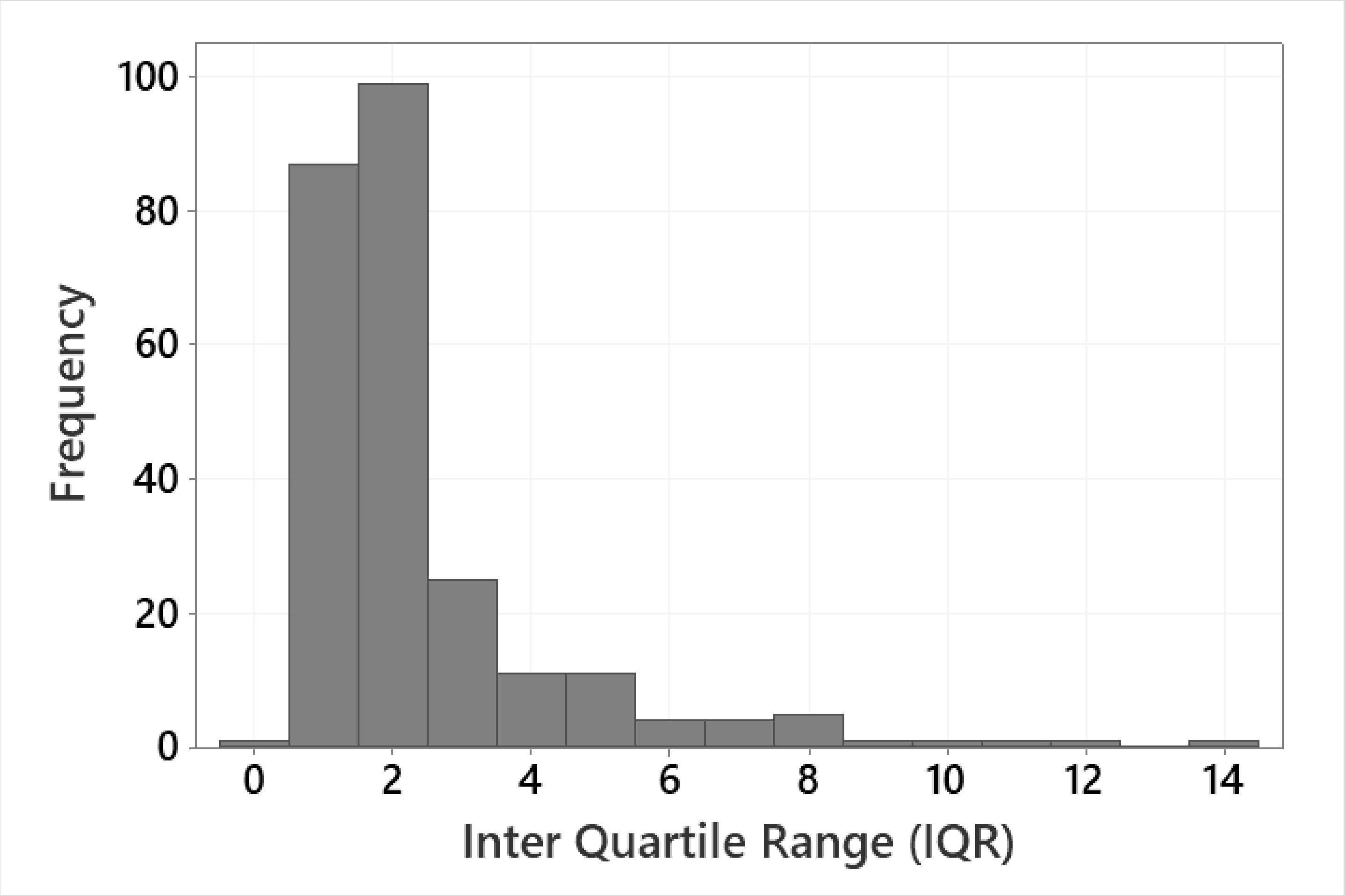

Supplemental Figure 2. Distribution of inter quartile range (IQR) for 247 miRNAs in 91 liver samples.

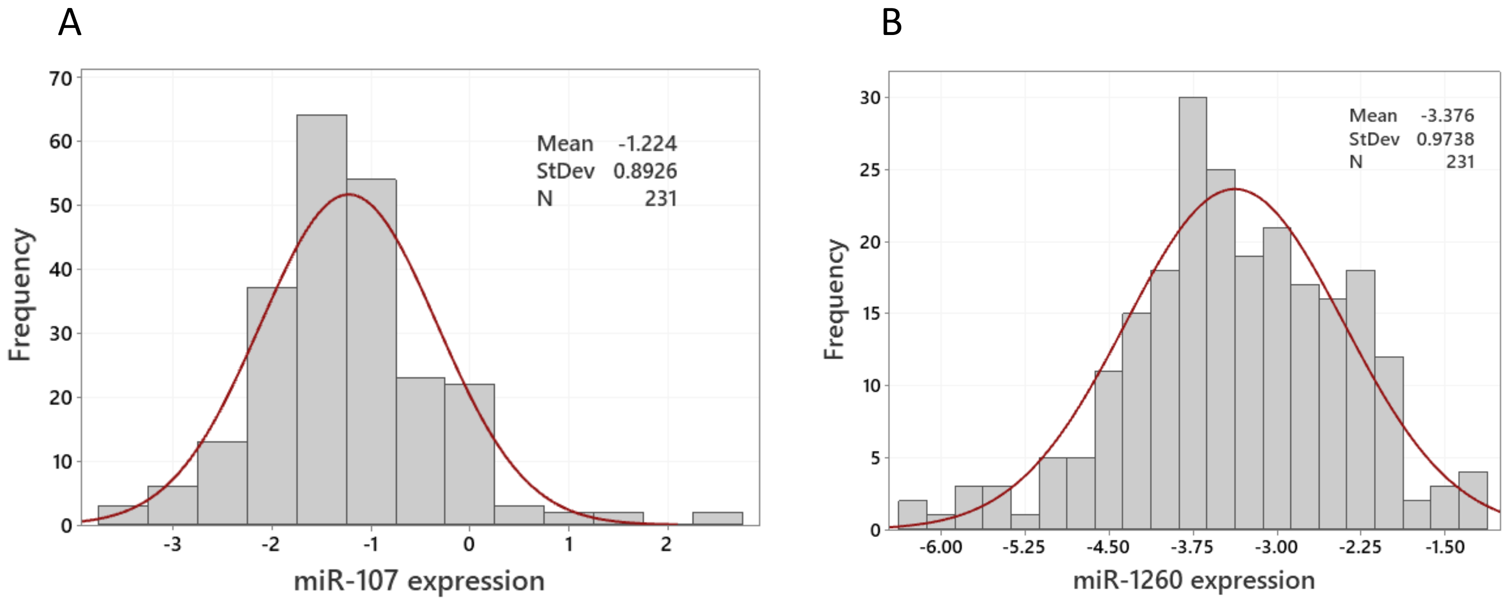

Supplemental Figure 3. Histogram of the expression levels of miR-107 (A) and miR-1260 (B) in 231 human liver samples.
